# Nanocomposite Films of Chitosan-Grafted Carbon Nano-Onions for Biomedical Applications

**DOI:** 10.3390/molecules25051203

**Published:** 2020-03-07

**Authors:** Carlos David Grande Tovar, Jorge Iván Castro, Carlos Humberto Valencia, Diana Paola Navia Porras, José Herminsul Mina Hernandez, Mayra Eliana Valencia Zapata, Manuel N. Chaur

**Affiliations:** 1 Grupo de investigación de fotoquímica y fotobiología, Universidad del Atlántico, Carrera 30 Número 8-49, Puerto Colombia 081008, Colombia; carlosgrande@mail.uniatlantico.edu.co; 2 Grupo de Investigación SIMERQO, Departamento de Química, Universidad del Valle, Calle 13 No. 100-00, Cali 76001, Colombia; jorge.castro@correounivalle.edu.co; 3 Escuela de Odontología, Grupo biomateriales dentales, Universidad del Valle, Calle 4B # 36-00, Cali 76001, Colombia; carlos.humberto.valencia@correounivalle.edu.co; 4 Grupo de Investigación Biotecnología, Facultad de Ingeniería, Universidad de San Buenaventura Cali, Carrera 122 # 6-65, Cali 76001, Colombia; dpnavia@usbcali.edu.co; 5 Escuela de Ingeniería de Materiales, Facultad de Ingeniería, Universidad del Valle, Calle 13 No. 100-00, Santiago de Cali 760032, Colombia; valencia.mayra@correounivalle.edu.co; 6 Centro de Excelencia en Nuevos Materiales (CENM), Universidad del Valle, Calle 13 No. 100-00, Santiago de Cali 760032, Colombia

**Keywords:** amide, biodegradable films, chitosan-grafted carbon nano-onions, poly (vinyl alcohol), tissue engineering

## Abstract

The design of scaffolding from biocompatible and resistant materials such as carbon nanomaterials and biopolymers has become very important, given the high rate of injured patients. Graphene and carbon nanotubes, for example, have been used to improve the physical, mechanical, and biological properties of different materials and devices. In this work, we report the grafting of carbon nano-onions with chitosan (CS-g-CNO) through an amide-type bond. These compounds were blended with chitosan and polyvinyl alcohol composites to produce films for subdermal implantation in Wistar rats. Films with physical mixture between chitosan, polyvinyl alcohol, and carbon nano-onions were also prepared for comparison purposes. Film characterization was performed with Fourier Transformation Infrared Spectroscopy (FTIR), Thermogravimetric Analysis (TGA), Differential Scanning Calorimetry (DSC), Tensile strength, X-ray Diffraction Spectroscopy (XRD), and Scanning Electron Microscopy (SEM). The degradation of films into simulated body fluid (SBF) showed losses between 14% and 16% of the initial weight after 25 days of treatment. Still, a faster degradation (weight loss and pH changes) was obtained with composites of CS-g-CNO due to a higher SBF interaction by hydrogen bonding. On the other hand, in vivo evaluation of nanocomposites during 30 days in Wistar rats, subdermal tissue demonstrated normal resorption of the materials with lower inflammation processes as compared with the physical blends of ox-CNO formulations. SBF hydrolytic results agreed with the in vivo degradation for all samples, demonstrating that with a higher ox-CNO content increased the stability of the material and decreased its degradation capacity; however, we observed greater reabsorption with the formulations including CS-g-CNO. With this research, we demonstrated the future impact of CS/PVA/CS-g-CNO nanocomposite films for biomedical applications.

## 1. Introduction

Shifting of the traditional paradigm of using synthetic polymers for the scaffold design to the uses of highly re-absorbable and compatible porous materials is happening in the last decade [1]. This new model needs the correct interconnected porosity and biocompatibility design and an interconnected three-dimensional (3D) structure with excellent mechanical properties resembling native tissue [1,2].

Scaffolds are three-dimensional structures with excellent capacity to promote cell adhesion, proliferation, and function differentiation [3]. Some studies have been undertaken to investigate the use of carbon-based nanomaterials for bone tissue engineering in vivo [4]. The new properties of carbon materials such as fullerene, CNT, and graphene have significantly increased their study and applications [5,6]. Due to their unique properties and high mechanical strength, they have been widely used to reinforce materials with biomedical applications and for tissue regeneration [7]. 

Natural polymers are “ahead of the race” to produce scaffolds due to their cell adhesion capacity and lower toxicity [8]. However, synthetic polymers have a lower commercial price, and the facile chemical functionalization facilitates cell adhesion improving biological responses [9]. Natural polymer limitations are often enhanced using composites with synthetic polymers are also prepared to improve hydrophilicity, cell attachment, and biodegradability [10,11]. Generally, for scaffold´s design, chitosan, alginate (Alg), collagen (Col), gelatin (Gn), silk fibroin (SF), and glycosaminoglycans (GAGs) are the most used biopolymers [11,12,13,14,15,16,17].

Chitosan (CS), a natural polymer, is one of the most common biopolymers applied in medical studies due to the biodegradability, low toxicity, and excellent resorption [18]. Tissue-engineering applications involving bone [19,20], cartilage [21,22], liver [23], tendons [24,25], ligaments and nerves [26,27], wound healing [28,29], separation membranes [30,31,32], blood anticoagulants [33,34,35,36], contact lenses [37], controlled release of drugs [38,39,40], fat-sequestering agent [41,42], hydrogel preparations [43,44], and food packaging material [45,46] were previously reported. However, CS has the typical drawbacks of a polysaccharide, such as low solubility and poor stability in physiological media due to hydrogen bonding [47].

The nanocomposite of CS and synthetic polymers have been prepared to overcome the limitation of naturally occurring polymers [47,48]. The application of carbon nanomaterials in tissue engineering is very appropriate, as carbon nanomaterials provide mechanical, thermal, antibiotic, and antifungal resistance in many cases, without generating tissue toxicity or immune system response [3,49].

Carbon nano-onions (CNO) are carbon structures containing multiple shells of fullerenes of 10 nm size. Ugarte discovered these nanomaterials in 1992 under the impact of carbon nanostructures with electrons through a TEM experiment [50]. For the chemical derivatization of CNO, various synthetic routes introducing new functional groups have been applied to add new properties and applications. Some potential uses include optical applications, catalysis, and gas storage [51].

However, the use of CNO for biomedical applications remains unexplored. There is a report in demand for the treatment of cancer [52] and the promotion of cell adhesion and proliferation through scaffolds in animal tissues without immune responses [53]. We previously reported nanocomposites of graphene oxide and chitosan with excellent compatibility and thermal improvement [54,55,56,57,58,59,60]. The synthesis of nanocomposites of chitosan/poly (vinyl alcohol)/oxidized carbon nano-onions (CNO/PVA/ox-CNO) was assessed and applied in Wistar rats’ subdermal tissues during 90 days, observing proper resorption with no immune response [61]. The membranes obtained in the previous work were very stable, especially in the in vivo subdermal implantation, even after 90 days. After that time, there was virtually no degradation in the membranes that included oxidized carbon nano-onions (ox-CNO). However, these membranes did not generate a severe inflammatory response or pus formation, so they were projected for long-term applications (bone tissue regeneration). On the other hand, we decided that if a synthetic modification was made on the nano-onions and they were grafted with chitosan directly, we could improve the biocompatibility and degradability of the material, which would allow a more significant resorption of the material thinking of projecting it for applications where degradation is required fast, such as dressing and skin applications for example.

In this research, chitosan was grafted with oxidized carbon nano-onions (ox-CNO) through an amide-type bond. CS-g-CNO was blended with chitosan and polyvinyl alcohol composites to produce films that, posteriorly, were subdermally implanted in Wistar rats. Films including CS/PVA/ox-CNO were also prepared whose mechanical, thermal, and chemical characterization was compared to those containing chitosan-grafted carbon nano-onions.

## 2. Results

### 2.1. CS-g-CNO Characterization

#### 2.1.1. Fourier-Transform Infrared Spectroscopy (FTIR)

**Figure 1:**
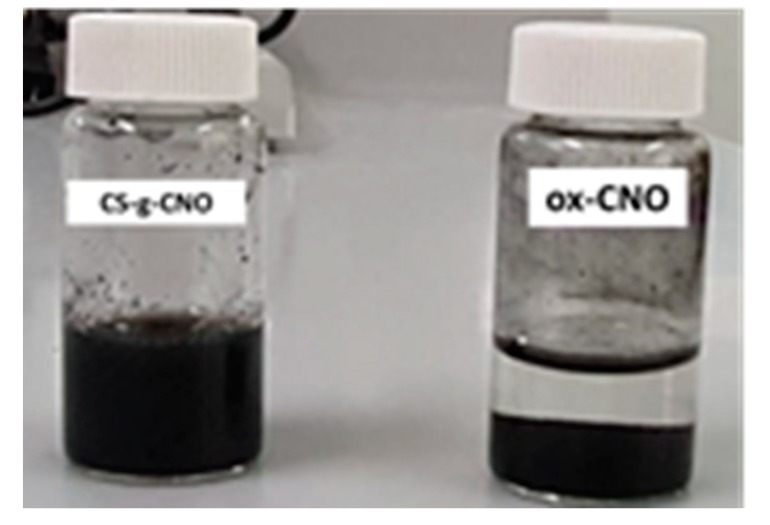
Image of ox-CNO and CS-g-CNO in 2% acetic acid dilution after 30 days.

**Figure 2:**
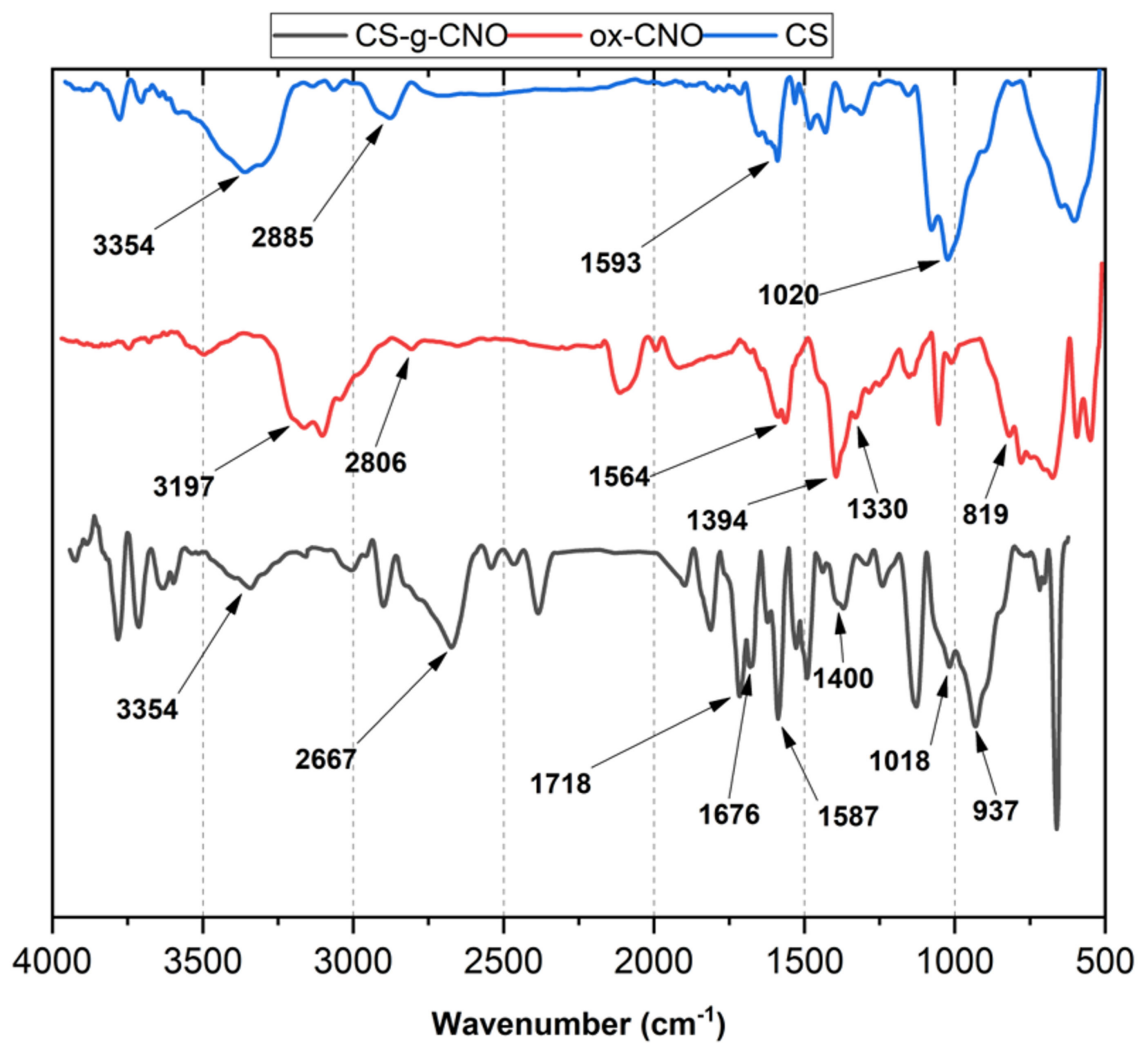
Attenuated total reflectance Fourier-transform infrared spectroscopy (ATR-FTIR) of CS, ox-CNO, and CS-g-CNO.

**Scheme 1:**
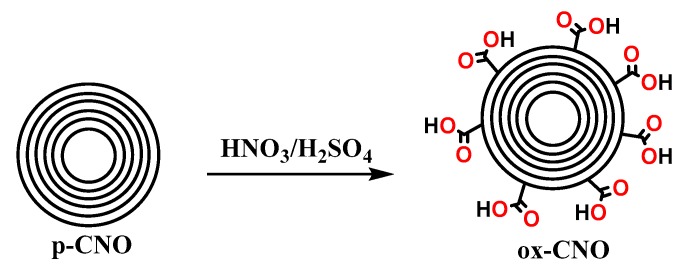
Preparation of oxidized carbon nano-onions (ox-CNOs) [85].

**Scheme 2:**
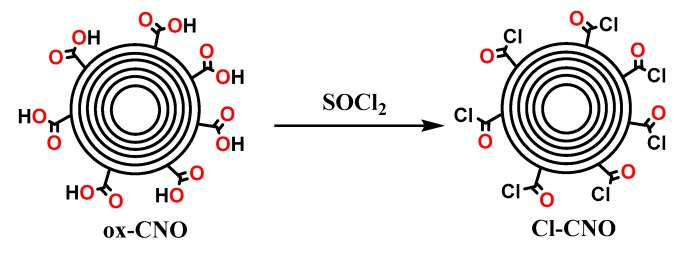
Synthesis of the acyl chloride-functionalized carbon nano-onions (Cl-CNO).

The functionalization of ox-CNO with chitosan is quite difficult due to the lack of reactivity of carboxylic groups with the amine groups [62]. However, to obtain covalent CS functionalization of CNO, it was necessary to convert ox-CNO to Cl-CNO as described in Scheme 1 and Scheme 2. The stability of the CS-g-CNO was evident after dispersion in dilute organic acids and long-term stability (Figure 1). Figure 2 shows the FTIR spectrum after the complete reaction between CS and Cl-CNO to produce the CS-g-CNO. For the ox-CNO, the leading bands present were broadband at 1730 cm^−1^ from the C=O stretching vibrations of carboxyl and carbonyl groups. The band at 1564 cm^−1^ also is due to the carboxyl and carbonyl groups [63]. Around 3200–3675 cm^−1^, a band related to adsorbed water for ox-CNO was observed. From the spectrum of the CS-g-CNO, the strong band at 1718 cm^−1^ resulted from the C=O stretching vibrations of carboxyl and carbonyl groups from the ester formation between acyl chloride groups of Cl-CNO (-ClCO-) and hydroxyl groups of CS forming ester bands (-COO-) also presenting broadband due to the C-stretching of esters at 1000 cm^−1^. The strong band at 1676 cm^−1^ is related to the secondary amide formed between the amine groups of chitosan (-NH_2_) and the acyl chloride groups of the precursor (-ClCNO-). The moderate band at 3354 cm^−1^ corresponds to the -NH stretching of amides. The strong band at 1587 cm^−1^ is related to -N-H bending of secondary amides after the formation of the bond with the CNO. Finally, the moderate group at 1400 cm^−1^ is associated with the C-N stretching of secondary amides [64]. Besides that, two characteristic bands of the glucopyranose rings appear at approximately 937 and 1018 cm^−1^, respectively, implying the attachment of the CS chains to the CNO as previously observed for the functionalization of carbon nanotubes with low molecular weight chitosan [65]. The new strong band noted at 1587 cm^−1^ corresponding to amide II, is clear evidence of the CNO functionalization with CS.

#### 2.1.2. X-ray Diffraction (XRD)

**Figure 3:**
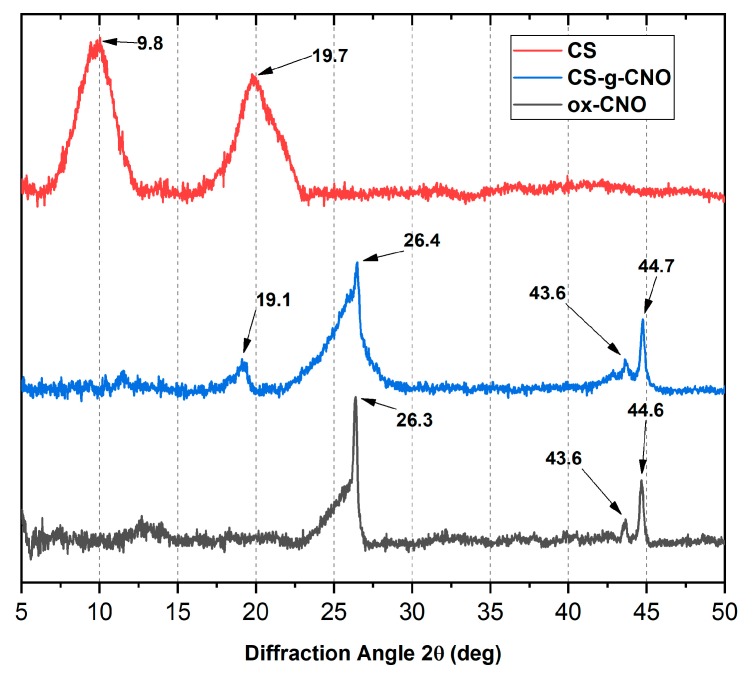
X-ray diffraction (XRD) analysis of CS (red line), CS-g-CNO (blue line), and ox-CNO (black line).

The crystal size and the interlayer distance of the inner layers of carbon nano-onions were determined by X-ray diffraction (XRD). X-ray diffraction patterns of CS, CS-g-CNO, and ox-CNO are observed in Figure 3. CS showed a peak at 2θ = 19.7° due to the crystalline structure [66]. The peak at 2θ = 9.8° was due to the amorphous region of CS. On the other hand, the broad peak at 43.6° for ox-CNO was related to the inter-planar distance in the crystal (100), while the sharp peak at 44.6°, corresponds to the reflection of the plane (111) of non-graphitized planes of traces of carbon nano-diamonds [61]. The peaks at 2θ = 26.3° and 44.6° are related to the (002) and (100) planes of the diffractions of the hexagonal graphite structure [66]. An average crystal size (τ) of 16.5 nm was calculated using Equation (2) and the peak (100). The average interplanar distance was calculated from the plane (002) at 26.4° (d) corresponding to 3.4 nm. Finally, using Equation (3), we determined a total of five-carbon shells by evaluation of 10% mass loss in a previous TGA [61]. 

For CS-g-CNO, peaks at 2θ = 26.4° and 44.7° from the CNO were evident, and almost no change in the interplanar distance inside the crystal was observed. However, at 2θ = 19.1°, there was a new peak corresponding to CS and which is related to its crystalline structure [66], demonstrating the grafting process on the surface of ox-CNO.

#### 2.1.3. Raman Spectroscopy

**Figure 4:**
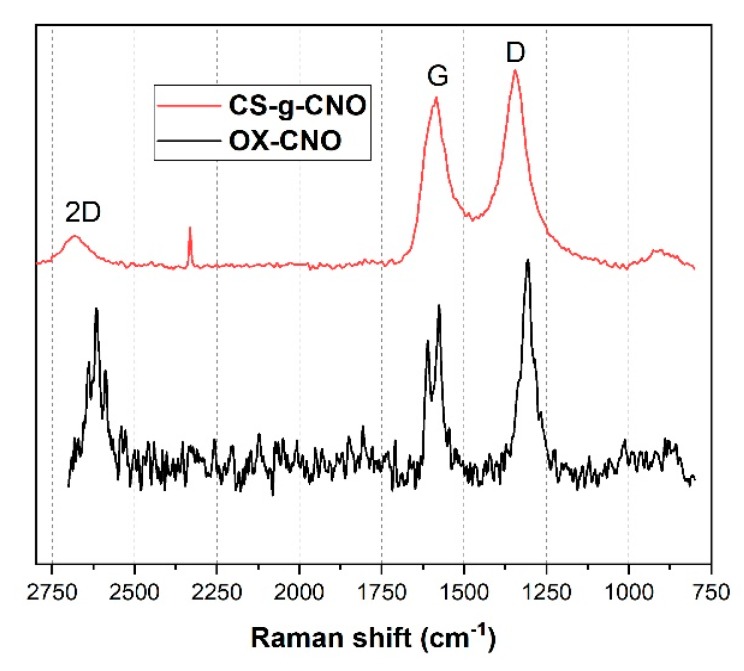
Raman spectrum of chitosan-grafted-carbon nano onions (red line) and oxidized carbon nano onions (black line).

Raman spectroscopy is an advantageous technique to characterize the graphitic structure of carbon nano-onion structures using two bands at 1300 and 1600 cm^−1^ (Figure 4). We could also distinguish the degree of order of the CNO utilizing the intensity of the ratio of *sp*^2^ and *sp*^3^ carbon atoms, which is related to the intensity and the shape of the D and G bands. Previously, we reported an increase for the ox-CNO structure the intensity of the D band related to the high amount of defects in the fullerene network due to the presence of oxygen-rich functional groups, as well as to an increase in the *sp*^3^ hybridized carbons because of the increasing disorder by oxygen functionalization [61]. Besides, the band at 2621 cm^−1^ was attributed to the 2D group of the highly ordered graphitic materials. On the other hand, it can be observed for the CS-g-CNO spectrum that D and G bands shifted and increased as a result of the covalent attachment of the chitosan to the acyl groups of the Cl-CNO. Chattopadhyay et al. also reported related results [67]. However, the 2D band showed the most noticeable changes as a result of the grafting method of the carbon structure with CS. As previously mentioned, this band is susceptible to changes in the structure of the allotropic carbon materials [68], and its broadening is related to the changes produced in the carbon nano-onions by the grafted chitosan.

#### 2.1.4. Thermogravimetric Analysis of CS-g-CNO

**Figure 5:**
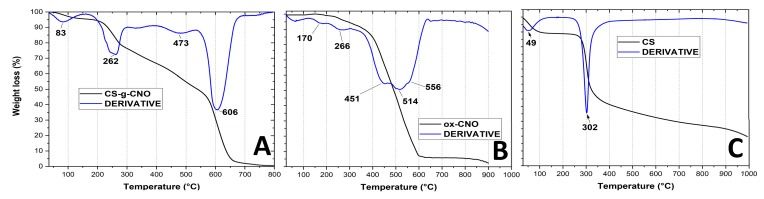
Thermogravimetric analysis of (**A**) chitosan-grafted carbon nano onion (CS-g-CNO), (**B**) oxidized carbon nano onion (ox-CNO), and (**C**) chitosan CS.

TGA is a useful technique to understand the degradation profile and the disorder degree of a compound. We previously studied the degradation process of ox-CNO and p-CNO and determined the average number of functional groups on the surface through XRD. With five shells, the number of carbons in each shell was calculated using Equation (4) [69]. The final result was a presence of 98 functional groups, around one every 15 (1500/98) carbons of the outer shell [61]. Figure 5 shows two decomposition temperatures corresponding to the carboxylic groups and hydroxyl groups at the CNO layer at 266 °C and 170 °C, respectively.

In the case of the TGA, the thermal analysis of pure chitosan shows two-weight losses. A 20% weight loss observed below 400 °C of the amine side or N-acetyl side groups of chitosan. This degradation appears to be delayed in CS-g-CNO. An indication that the presence of carbon nano-onions in the chitosan enhanced the thermal stability in chitosan. The second weight loss occurs between 600 and 700 °C due to the decomposition of the carbon nano-onion structure and glycoside fragments of the chitosan [70].

### 2.2. Film Characterization

#### 2.2.1. Fourier-Transform Infrared Spectroscopy (FTIR)

**Figure 6:**
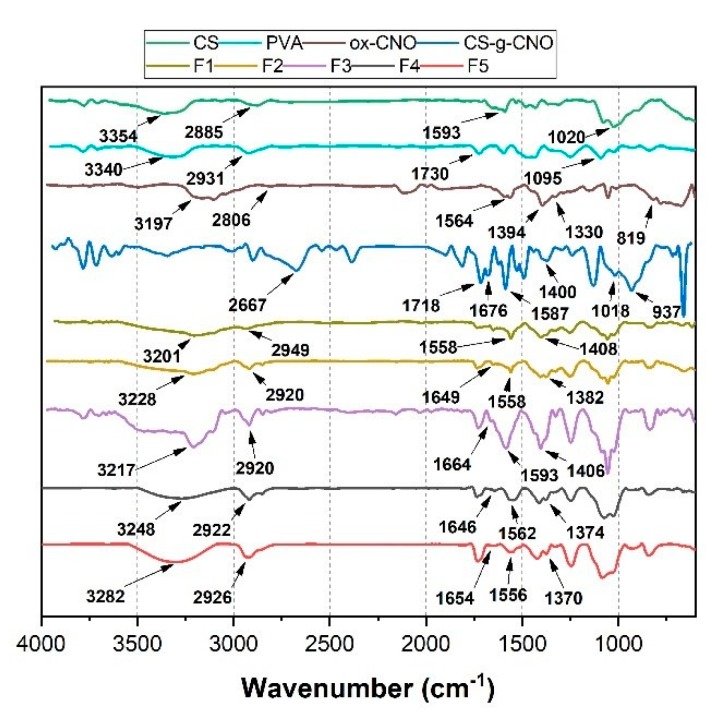
Attenuated total reflectance Fourier-transform infrared spectroscopy (ATR-FTIR) of the formulations: F1 (CS:PVA:ox-CNO 30.00:70.00:0); F2 (CS:PVA:ox-CNO 29.50:70.00:0.50); F3 (CS:PVA:OX-CNO 29.00:70.00:1.00); F4 (CS:PVA:CS-g-CNO 29.50:70.00:0.50); and F5 (CS:PVA:CS-g-CNO 29.00:70.00:1.00).

Figure 6 shows the FTIR spectrum of the nanocomposite films. For F1 PVA and CS, characteristic bands are observed [71]. At 1640 and 1560 cm^−1^, -NH-C=O stretching bands were found. Chitosan bands shifted due to the hydrogen bonding with PVA. Around 3000–3500 cm^−1^, broadband-related to hydroxyl and amine groups is found, with an evident shift due to the hydrogen bonding. CS/PVA composite films (F1) had bands also between 3201 and 2949 cm^−1^ due to –OH and –CH_2_ from PVA [72].

The bands at 1647, 1558, and 1327 cm^−1^ are related with amides I and III of C=O stretching vibration, N–H bending of NH_2_, and CH_2_ wagging and also OH groups from CS while the peak at 1408 cm^−1^ is related to OH and CH groups [73]. F2 and F3 composite film spectra presented a new band at 1750 cm^−1^ due to –COOH groups from ox-CNO. On the other hand, for the CS/PVA/CS-g-CNO composites (F4 and F5), the band at 3248 for amine groups of CS overlapped the -NH stretching of amides band of CS-g-CNO. The strong band at 1646 cm^−1^ was related to the carbonyl (-C=O) amide (-NHCO-) between the amine groups of chitosan (-NH_2_) and the acyl chloride CNO precursor (-ClCNO). The moderate band at 3354 cm^−1^ was related to the -NH stretching of amides. The strong group at 1562 cm^−1^ was also related to -N-H bending of secondary amides. The group at 1374 cm^−1^ was related to the C-N bond of amides from chitosan.

#### 2.2.2. X-ray Diffraction (XRD)

**Figure 7:**
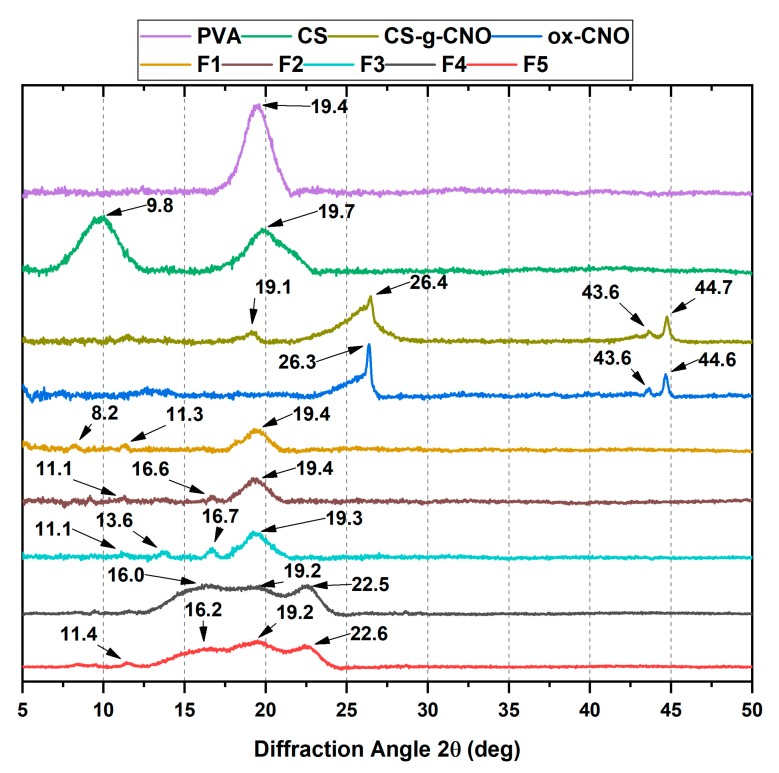
X-ray diffraction (XRD) of the different nanocomposite films. Formulations: F1 (CS: PVA:ox-CNO 30.00:70.00:0); F2 (CS:PVA:ox-CNO 29.50:70.00:0.50); F3 (CS:PVA:ox-CNO 29.00:70.00:1.00); F4 (CS:PVA:CS-g-CNO 29.50:70.00:0.50); and F5 (CS:PVA:CS-g-CNO 29.00:70.00:1.00).

The crystallinity of the nanocomposite films is shown in Figure 7. The XRD pattern of the F1 showed two diffraction peaks at 2θ = 8.2° and 11.3° produced from the amorphous structure of CS [61]. The broad peak at 2θ = 19.4° was related to the PVA crystals [74]. The hydrogen bond formation shifted the position of several peaks. For F2 and F3 composite film spectra (CS/PVA/ox-CNO), three new peaks at 2θ = 11.1°, 13.6°, and 16.6° were observed as a result of the ox-CNO physical mixture. For the F4 and F5 composites including CS-g-CNO, vast peaks at 2θ = 11.4°, 16.2°, 19,2°, and 22.6° were observed, as a result of the changes in the crystalline structure due to the presence of CS-g-CNO and CS/PVA with strong hydrogen bonding between the polymer chains. At 2θ = 22.6° the peak corresponding to the (002) plane of CNO was shifted while the peaks at 2θ = 19.2° were related to the PVA crystals. Peaks at 2θ = 11.4° and 2θ = 16.2° were associated with the presence of the CNO core [66]. 

#### 2.2.3. Scanning Electron Microscope (SEM)

**Figure 8:**
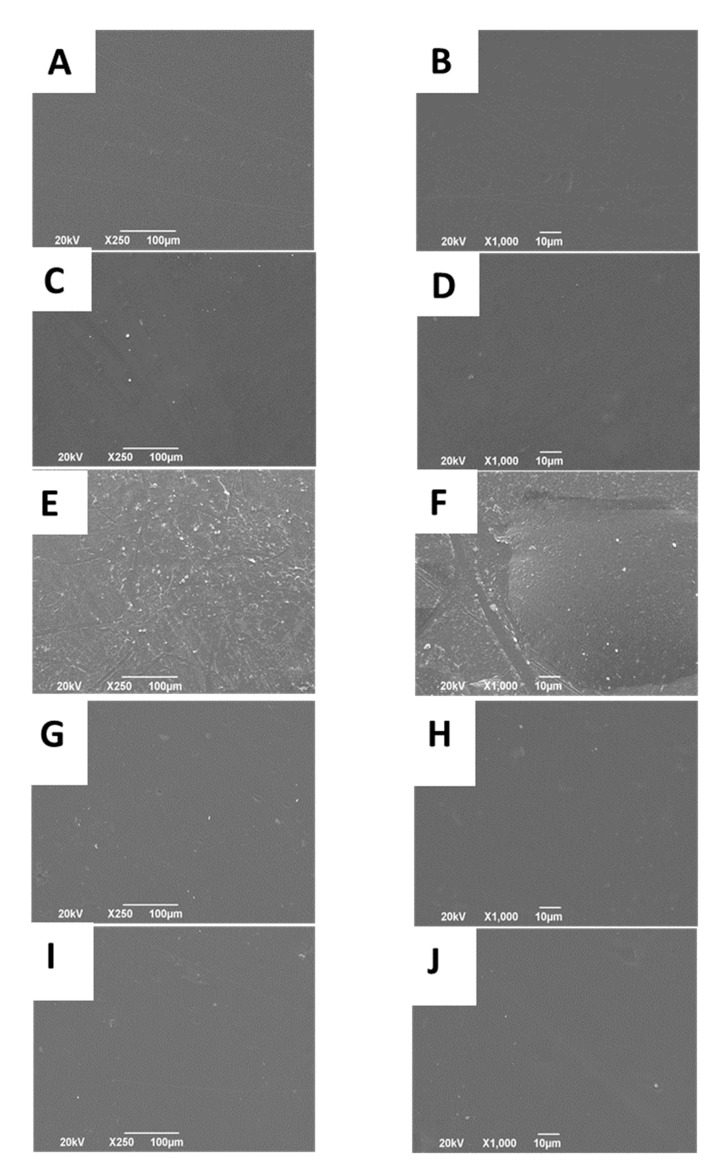
Morphology of the films: F1 (CS: PVA:ox-CNO 30.00:70.00:0) (**A**) at 250×, (**B**) at 1000×; F2 (CS:PVA:ox-CNO 29.50:70.00:0.50) (**C**) at 250×, (**D**) at 1000×; F3 (CS:PVA:ox-CNO 29.00:70.00:1.00) (**E**) at 250×, (**F**) at 1000×; F4 (CS:PVA:CS-g-CNO 29.50:70.00:0.50) (**G**) at 250×, (**H**) at 1000×; F5 (CS:PVA:CS-g-CNO 29.00:70.00:1.00) (**I**) at 250×, and (**J**) at 1000×.

The surface morphology of the nanocomposite films is presented in Figure 8. Figure 8A,B the high amount of hydrogen bonding between CS/PVA produces a crystalline, smooth, and homogeneous surface [56]. Nanocomposites from physical mixtures (Figure 8C–F) became rough and irregular due to ox-CNO presence between CS-PVA chains. By adding ox-CNO to the CS/PVA blend, the nanomaterial increased the roughness of the films due to their texture and phase separation [72]. The higher amount of opacity and darkening is a result of the bigger amount of ox-CNO [75]. When the composites were produced introducing CS-g-CNO (CS/PVA/CS-g-CNO 0.5% and 1.0%), the surfaces become smoother as compared to the nanocomposites with a physical mixture of ox-CNO. Better compatibility between the CS/PVA composite could be the result of hydrogen bonding with amide groups of CS-g-CNO, which will help the formation of a more homogeneous surface. More hydrogen bonding is possible with the grafting functionalization of carbon nano-onions, which is also an advantage for dispersion in body fluids and reabsorption.

#### 2.2.4. The Tensile Strength of the Films

Materials used for scaffold designs should have improved thermal resistances [72]. Film thicknesses were in the range of 62.3–80.8 μm for the films, and those thicknesses were used in the mechanical property’s tests. Young’s modulus and the tensile strength of the nanocomposites are presented in Table 1. For Young’s modulus, it is obvious that the introduction of the ox-CNO to the CS/PVA matrix, no significant (*p* < 0.05) differences were presented. However, with the introduction of the CS-g-CNO to the CS/PVA matrix, a significant (*p* < 0.05) decrease in Young’s modulus occurred, which is following an easier hydrolytic degradation that occurred. The increase in the CS-g-CNO in CS/PVA matrix (F5) significantly increased Young’s modulus as compared to F4. This could be the result of a stronger interaction with the CS/PVA matrix by hydrogen bonding and a higher dispersion, following the microstructure analysis. Figure S1 (Supplementary Materials) and Table 1 show a significant decrease in the tensile strength for F4 and F5 as compared to F1–F3. The introduction of the CS-g-CNO instead of ox-CNO might not have the same reinforcement effect due to a change in the crystallinity of the CNO and CS, as observed in the XRD analysis. Between F4 and F5, there were no significant (*p* < 0.05) differences in the tensile strength. Introduction of 1.0 wt % ox-CNO to the CS/PVA matrix slightly increased the tensile strength up to 43.1 ± 0.77 GPa. The increasing of the tensile strength is a result of a more balanced distribution of the stress employing a more homogeneous structure produced from a proper dispersion of the nanomaterial in the polymer matrix [76]. It is possible that, as the amount of the nanomaterial increased, better dispersion and improvement of the mechanical properties occurs. With the introduction of the CS-g-CNO (with covalent functionalization) due to the chemical modification of the CNO, it could lose mechanical resistance. The previous result agrees with the faster hydrolytic degradation in SBF and in vivo reabsorption results. Young’s modulus decreased up to 2.26 ± 0.15 GPa and the tensile strength to 28.0 ± 2.27 MPa for the formulation F5 (CS:PVA: CS-g-CNO 29.00:70.00:1.00).

#### 2.2.5. Thermal Studies

The thermogravimetric analysis (TGA; Figure S2, Supplementary Materials) includes three mayor decomposition stages for the nanocomposites. Below 200 °C free water and more volatile compounds were lost as a result of the heating process. Between 200 and 400 °C the degradation of PVA and CS weaker bonds occurred. The final stage above 410 °C for F1 (CS/PVA) and 423 °C for F3 (CS:PVA:ox-CNO 29.00:70.00:1.00) was attributed to the decomposition of the nano-onion structure [72]. The ox-CNO incorporation into the composite CS/PVA to get a physical mixture increased the degradation temperatures (Table 1). The Td_3%_ (3% mass loss) of the composites was exhibited in Table 1. Td_3%_ for the composites, including CS-g-CNO, demonstrated a decrease in Td_3%_, as shown in Table 1. At 600 °C, the composite was also wholly degraded, demonstrating a more homogeneous material, due to the chemical functionalization of the CNO. With the physical mixture composites (F2 and F3), 10 wt % remained after 1000 °C, which indicated a high thermal stability material. With the incorporation of CS-g-CNO even at 0.5 wt %, lower stability is presented by the composites starting from 323 °C the degradation.

Differential Scanning Calorimetry (DSC) analysis is useful to understand the thermal behavior of the nanocomposites better. The glass transition temperature (T_g_) of F3 (ox-CNO 1.0 wt %) increased from 19.9 (F1) to 79.3 °C, which also demonstrated a thermal reinforcement of the CS/PVA composite (Table 1). For the composites, including CS-g-CNO (F4 and F5), lower Tg was obtained and decreasing with the increasing of the CS-g-CNO, indicating that lower thermal stability was present and a more-likely structure to the F1, due to the higher compatibility between CS/PVA and CS-g-CNO. Grafting of CNO with CS will help the CNO to disperse better into the CS/PVA matrix due to the higher chemical compatibility, creating a similar structure to the original CS/PVA. 

#### 2.2.6. Degradation in a Simulated Biological Fluid (SBF)

##### Weight Loss

**Figure 9:**
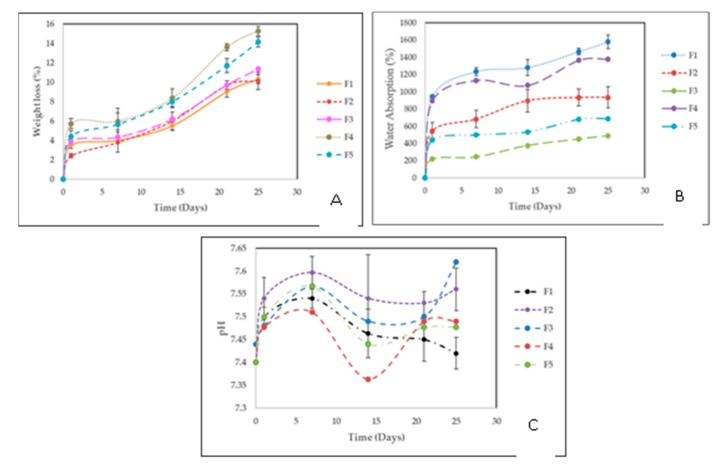
(**A**) Weight loss vs. immersion time in a simulated biological fluid (SBF) of the films. (**B**) Water absorption vs. immersion time in a simulated biological fluid (SBF) of the films. (**C**) Study of the solution pH vs. immersion time in a simulated biological fluid (SBF) of the films. Formulations: F1 (CS: PVA:ox-CNO 30.00:70.00:0); F2 (CS:PVA:ox-CNO 29.50:70.00:0.50); F3 (CS:PVA:ox-CNO 29.00:70.00:1.00); F4 (CS:PVA:CS-g-CNO 29.50:70.00:0.50); and F5 (CS:PVA:CS-g-CNO 29.00:70.00:1.00).

Figure 9A shows the results of the degradation percentage (weight loss) of the films during 25 days of immersion in SBF. The incorporation of ox-CNO in the polymeric matrix did not considerably affect the stability of the films since the weight loss was very similar for F1, F2, and F3. A slight increase in the water loss could be the result of a higher porosity due to the incorporation of ox-CNO, separation of the hydrogen bonding of CS and PVA chains, or ox-CNO hydrophilic groups. However, formulations, including CS-g-CNO, allowed a higher weight loss presumably due to higher hydrogen bonds with water and a loss of crystallinity of the polymer structure, facilitating the water hydrolysis. This higher weight loss could be an advantage in biomedical applications where fast reabsorption is needed, like orthopedic and subdermal applications. However, higher amounts of CS-g-CNO decreased the weight loss, maybe due to the higher quantity of CNO incorporated, which also is an agreement with the thermal and mechanical test results.

##### Water absorption

Body fluid absorption supports cell–biomaterial interactions and reabsorption of the material [60]. Water absorption was significant for the films since it allowed the interaction with the SBF and shows if that interaction would affect the stability of the films. Water absorption of CS/PVA films would allow the formation of a fibrous membrane around the films once the material is subdermally implanted. From Figure 9B, it can be observed that when the samples were immersed in the simulated body fluid (SBF), the absorption of water increased with the immersion time. The film composites of CS/PVA (F1) had higher water uptake after 25 days, presumably due to the higher hydrogen bonding with water and higher hydrophilicity of CS [77]. F2 and F3 ox-CNO (0.5 wt % and 1.0 wt %) had low water uptake due to the ox-CNO presence as compared to F4 and F5. F4 and F5 introduced CS/PVA/CS-g-CNO, which have new amide and ester groups that support a higher amount of hydrogen bonds with the water, which also supports a better SBF interaction. We could conclude that the chemical functionalization facilitates biomaterial–water communication and reabsorption of the material, which also will be useful in biomedical applications where a fast degradation could be necessary, like in orthopedic [78].

##### pH Changes

Figure 9C shows the pH changes during 25 days of immersion of nanocomposites in SBF. The formation of acid species after several days of film immersion is a hydrolytic degradation of the amorphous zones of the polymers, especially for F1, F4, and F5, where chitosan is more susceptible to deterioration and a higher affinity of water is present [79]. The reduction of the pH is a result of the detachment of CS by-products like glucosamine and *N*-acetylglucosamine [61]. These by-products usually are present in the extracellular matrix of humans tissues, which are not dangerous for human health [80]. Degradation is possible since a biological pH media (between 6 and 8) supports several cell enzymatic reactions [56,59,81]. Very importantly, the final pH after degradation for all the nanocomposites was within the permissible range in the human body to promote the life and maintenance of vital functions.

#### 2.2.7. Biomodel In Vivo Tests

**Figure 10:**
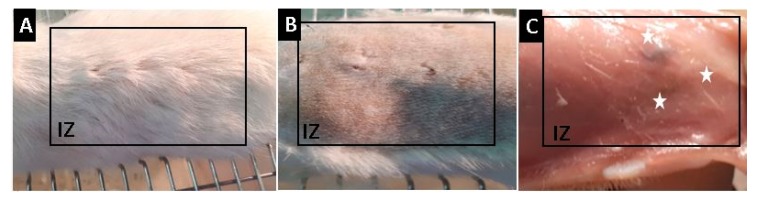
Macroscopic appearance of the Wistar’s rat skin implanted. (**A**): Formation of new skin. (**B**): Skin after shaving. (**C**): Subdermal appearance of the skin. **IZ:** Implanted zone. **Stars**: Implanted material.

After thirty days of implantation, euthanasia of the models was performed, and the samples were recovered. Complete hair recovery was observed in the implanted area. Trichotomy (shaving) was accomplished, following the skin without continuity solutions and with healthy healing without signs of inflammation. On the other hand, a longitudinal incision was made, and the skin was utterly separated to visualize the implanted sites finding small areas where implanted materials were included, without signs of inflammation or infection such as redness, the presence of purulent exudate, or bad smell. Figure 10 corresponds to the macroscopic appearance of the skin of bio models at 30 days of implantation

**Figure 11:**
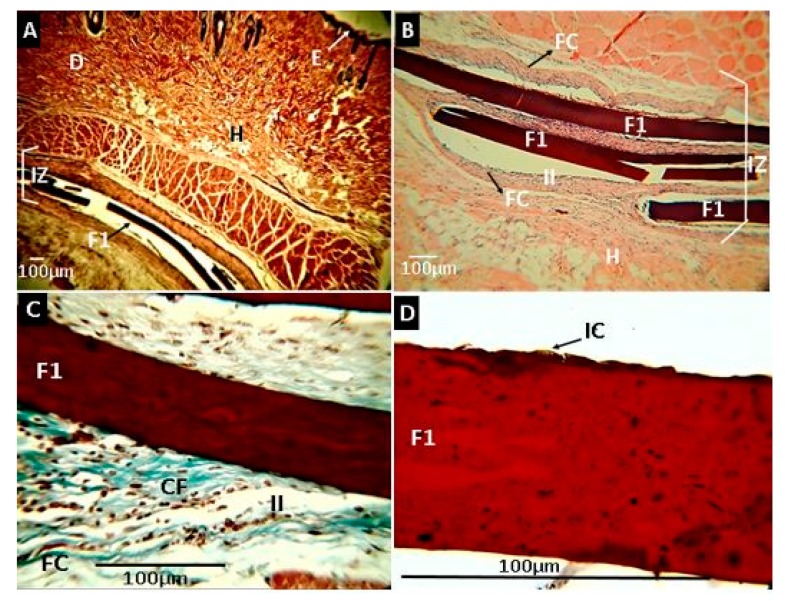
Histological analysis of experimental sample F1 (CS: PVA:ox-CNO 30.00:70.00:0). Magnifications are as follows: (**A**) 4× H&E, (**B**) 10× H&E, (**C**) 40× Masson’s trichrome stain (MT), (**D**) 100× MT. E: epidermis. D: dermis. H: hypodermis. IZ: implanted zone. II: Inflammatory infiltrate. FC: Fibrous capsule. CF: Collagen fibers type I. IC: Inflammatory cells. F1: Film formulation (CS: PVA:ox-CNO 30.00:70.00:0), 1. Hematoxylin and eosin (H&E) and Masson’s trichrome stain (MT) techniques.

Figure 11 corresponds to the analysis of the film’s subdermally implanted with formulation F1 after 30 days of implantation. In general, it was observed that the material showed no evidence of reabsorption. In the implantation area (IZ), the material was in a capsule (FC) containing an inflammatory cell infiltrate (II). Inflammatory cells (IC) were observed on the surface of the material. The capsule was made up of blue type I (CF) collagen fibers, evidenced by hematoxylin and eosin (H&E) and Masson’s Trichrome stain (MT). The inflammation process was typical for this type of subdermal process, without the presence of redness or pus, which demonstrated excellent compatibility with the tissue.

Chitosan degradation is a result of the phagocytosis and enzymatic activity of cells in subdermal implantation. Degradation is a function of the properties of the chitosan, like deacetylation degree, molecular weight, and cross-linking degree, among others [82,83]. Chitosan biocompatibility was demonstrated and the natural degradation, inducing tissue recovering. For example, Fujita et al. (2004) synthesized chitosan hydrogels that, after 20 days of subdermal implantation, were reabsorbed [84]. 

**Figure 12:**
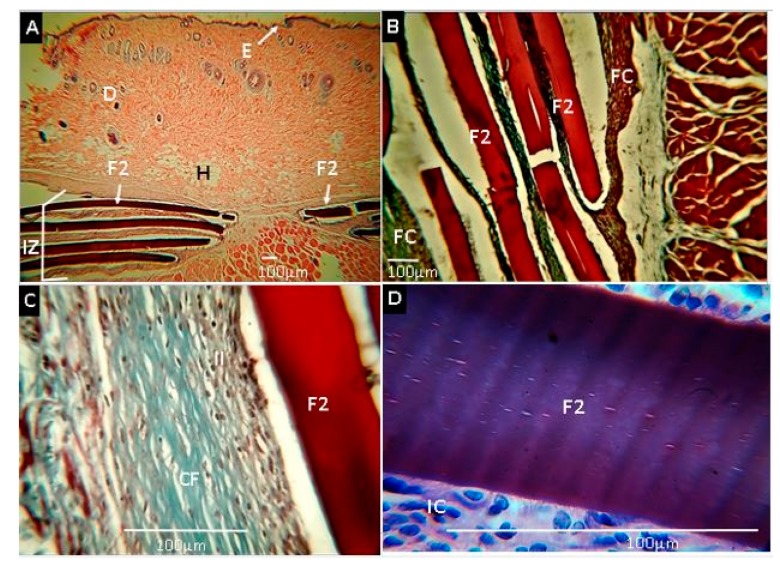
Histological analysis of experimental sample F2 (CS:PVA:ox-CNO 29.50:70.00:0.50). Magnifications are as follows: (**A**) 4× H&E, (**B**) 10× H&E, (**C**) 40× MT, (**D**) 100× MT. E: epidermis. D: dermis. H: hypodermis. IZ: implanted zone. II: Inflammatory infiltrate. FC: Fibrous capsule. CF: Collagen fibers type I. IC: Inflammatory cells. F2: Film formulation 2: (CS:PVA:ox-CNO 29.50:70.00:0.50). Hematoxylin and eosin (H&E) and Masson’s trichrome stain (MT) techniques.

Formulation F2 (CS:PVA:ox-CNO 29.50:70.00:0.50) exhibited similar behavior (healthy healing) after 30 days of implantation characterized by little reabsorption or degradation and being surrounded by a fibrous capsule composed of type-I collagen fibers (Figure 12). An inflammatory cell infiltrate was observed inside the capsule and in contact with the surface of the films. However, no immune response or pus production was observed, indicating a compatible material with the tissue.

**Figure 13:**
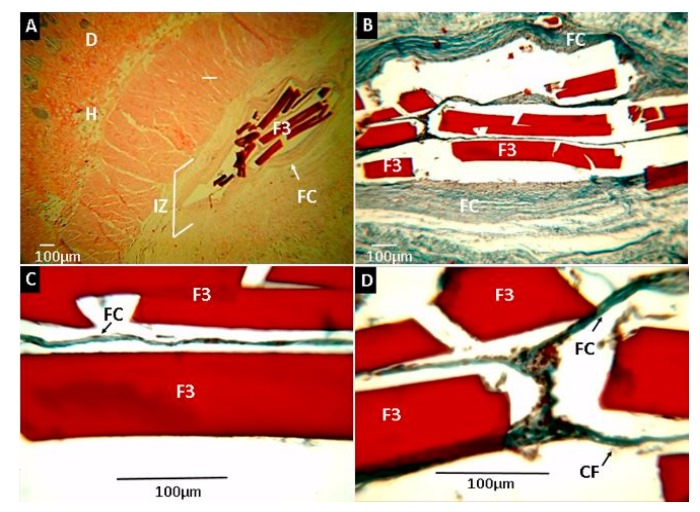
Histological analysis of experimental sample F3 (CS:PVA:ox-CNO 29.00:70.00:1.00). Magnifications are as follows: (**A**) 4× H&E, (**B**) 10× H&E, (**C**) 40× MT, (**D**) 100× MT. D: dermis. H: hypodermis. IZ: implanted zone. FC: Fibrous capsule. CF: Collagen fibers type I. F3: Film formulation 3 (CS:PVA:ox-CNO 29.00:70.00:1.00). Hematoxylin and eosin (H&E) and Masson’s trichrome stain (MT) techniques.

Samples of formulation F3 are observed in Figure 13. Although no degradation was seen on the surface, reabsorption at the extremes was shown in one of the samples (Figure 13D), demonstrating high compatibility with rat tissue, without causing allergic reactions or pus formation.

Nanocomposites of CS/PVA, including CS-g-CNO (F4 and F5), were surrounded by a fibrous capsule consisting of type I collagen, but unlike the above, more significant material degradation was observed.

**Figure 14:**
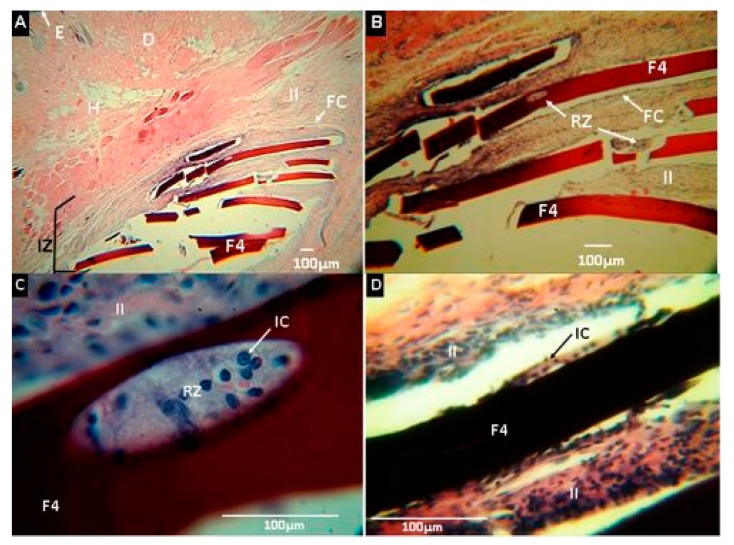
Histological analysis of experimental sample F4 (CS:PVA: CS-g-CNO 29.50:70.00:0.50). Magnifications are as follows: (**A**) 4× H&E, (**B**) 10× H&E, (**C**) 40× MT, (**D**) 100× MT. E: epidermis. D: Dermis. FC: Fibrous capsule. II: Inflammatory infiltrate. RZ: Reabsorption zone. IC: Inflammatory cells. F4: Film formulation 4(CS:PVA:CS-g-CNO 29.50:70.00:0.50). Hematoxylin and eosin (H&E) and Masson’s trichrome stain (MT) techniques.

Figure 14 corresponds to the F4 formulation, in which reabsorption was seen at the ends of the samples (Figure 14B) and on its surface with phagocytic activity by inflammatory cells (Figure 14C).

**Figure 15:**
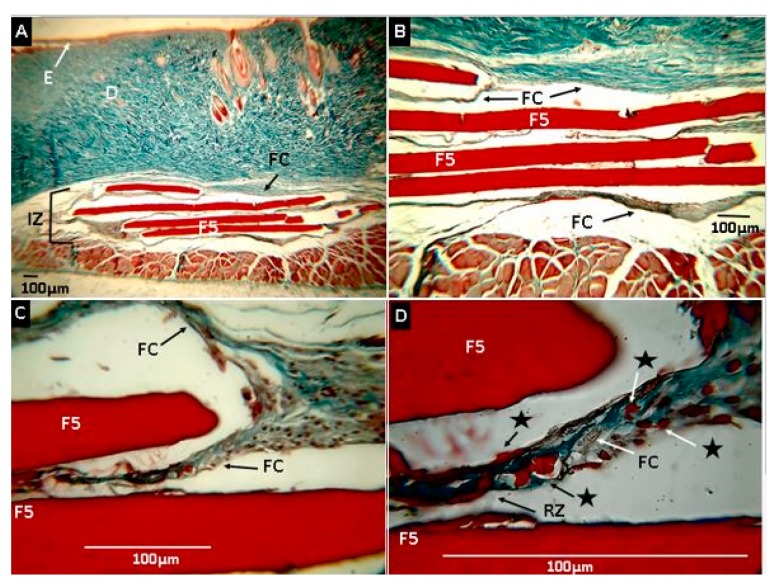
Histological analysis of experimental sample F5 (CS:PVA: CS-g-CNO 29.00:70.00:1.00). Magnifications are as follows: (**A**) 4× H&E, (**B**) 10× H&E, (**C**) 40× MT, (**D**) 100× MT. E: epidermis. D: Dermis. FC: Fibrous capsule. IZ: implanted zone. RZ: Reabsorption zone. Stars: Fragments of material being reabsorbed. F5: Film formulation 5 (CS:PVA:CS-g-CNO 29.00:70.00:1.00). Hematoxylin and eosin (H&E) and Masson’s trichrome stain (MT) techniques.

Samples obtained with the F5 formulation showed increased evidence of resorption/degradation (Figure 15). Figure 15C,D shows rounded ends by resorption activity with material fragments included in the fibrous capsule. The higher magnification in the fibrous capsule surrounding the material, small pieces of films (marked with stars), and an inflammatory infiltrate, responsible for phagocytosis, were seen. This result shows that the covalent functionalization of nano-onions with chitosan made them much more compatible with rat tissue, allowing for more considerable degradation and resorption. This result appears to be dependent on the amount of CS-g-CNO included in the nanocomposite, as the higher the amount, the greater the resorption. The higher compatibility with the rat’s tissue could be a result of the introduction of amide groups and chitosan to the CNO, because of a higher hydrogen bond will be present with this compound that using only ox-CNO in the CS/PVA matrix. Previously, our group demonstrated that after 90 days of subdermal implantation, CS/PVA/ox-CNO nanocomposites were compatible but with low resorption percentage [61]. In the present study, we demonstrated that carbon nano-onions grafting with chitosan improved the biocompatibility and resorption of the films, a fact that stimulated tissue regeneration.

## 3. Materials and Methods 

### 3.1. Materials

The synthesis of ox-CNO was already reported elsewhere [61]. Nanocomposite films were produced using chitosan of low molecular weight (molecular weight (Mw.) 144,000 g/mol, deacetylation degree 89–90%), poly(vinyl alcohol) (PVA; hydrolysis degree 87–89%, Mw. 93,000 g/mol, Sigma-Aldrich, Palo Alto, CA, USA). CS solutions were prepared using glacial acetic acid from Merck (Burlington, MA, USA). Simulated biological fluid (SBF) was prepared using NaCl, K_2_HPO_4_·3H_2_O, CaCl_2_, Na_2_SO_4_, and tris-(hydroxymethyl aminomethane) ((CH_2_OH)_3_CNH_2_) acquired from Sigma Aldrich (Palo Alto, CA, USA), a well as NaHCO_3_, KCl, and MgCl_2_·6H_2_O from Fisher Chemical (Pittsburgh, PA, USA), and hydrochloric acid (HCl) from Merck (Burlington, MA, USA). All reagents were analytical grade and used without any purification unless otherwise stated.

### 3.2. Methods

#### 3.2.1. Synthesis of ox-CNO, Cl-CNO, and CS-g-CNO

Oxidized carbon nano-onions (ox-CNO) were prepared using the previously reported methodology [61]. Briefly, using 40 mL of a 1:3 mixture of H_2_SO_4_: HNO_3_ and 500 mg of pristine carbon nano-onions (p-CNO) under reflux for five hours (Scheme 1). The impurities were removed by centrifugation and exhaustive washing with water, methanol, and ethanol [85].

The next step was the acyl chloride-functionalized CNO (CNO-COCl, Scheme 2) preparation based on a similar synthetic modification performed for multi-walled carbon nanotubes (MWCNT) [86], which was achieved by suspending the ox-CNO in a solution of thionyl chloride (5 mL). The suspension was stirred for 24 h at 70 °C. Then, a black solid was separated after solvent removal was half-reduced under reduced pressure on a rotary evaporator (Heidolph Hei-VAP Value Digital HB/G3B with vertical coil condenser, Germany) and then dried on a vacuum oven overnight. 

**Scheme 3:**
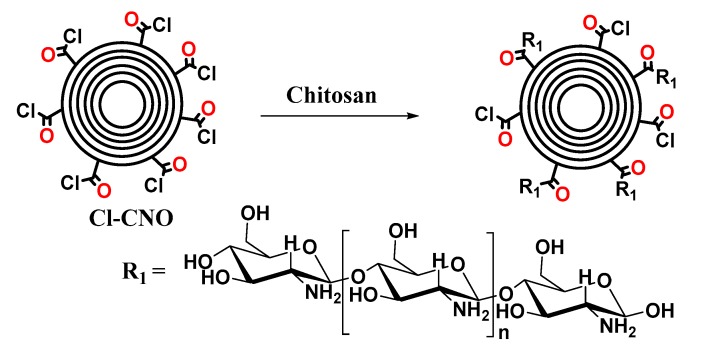
Preparation of chitosan-grafted carbon nano-onions (CS-g-CNO).

The final product corresponding to the chitosan grafting with oxidized carbon nano-onions (CS-g-CNO, Scheme 3) was obtained, adding 500 mg of Cl-CNO and 2 g of chitosan in 100 mL of acid acetic of 1% (*v/v*). The mixture was stirred at 100 °C for 48 h. After that, the combination was centrifuged, and three washes removed the impurities with a solution of the 1% solution of acetic acid.

#### 3.2.2. Characterization of ox-CNO and CS-g-CNO

FTIR characterization of ox-CNO and CS-g-CNO was carried out in an IR Affinity-1 infrared spectrophotometer (Shimadzu, Kyoto, Japan) in a range of 500–4000 cm^−1^ in the transmittance mode using the diamond tip method.

The degree of deformation and oxidation of the different carbon nano-onions were analyzed with a ThermoScientific X-ray diffraction (XRD) (Thermo Scientific, Waltham, MA, USA) Smart Raman laser beam 532 nm.

The X-ray powder diffraction analysis of the carbon nano-onions were taken on a PANalytical X’Pert PRO diffractometer (Malvern PANalytical, Jarman Way, Royston, UK) using Cu Kα1 radiation (1.540598 Å) and Kα2 (1.544426 Å), with an electron accelerator voltage of 45 kV, an electron-generating current of 40 mA, an optical grid of incident beam 1°, and a diffracted beam grid of 9.1 mm, in a range 2θ between 5 and 50°. The crystal size and the interlayer distance were calculated using the Bragg law (Equation (1)) and the Scherrer (Equation (2)) equations.

(1) $$d=\frac{\lambda }{2sen\theta }$$

(2) $$\tau =\frac{K\lambda }{\beta cos\theta }$$

For the Scherrer equation (Equation (2)), λ is the X-ray wavelength, k is a dimensionless shape factor with a value of 0.9, and β is the full width at half maximum in radians.

On the other hand, the number of shells of the oxidized carbon nano onions was previously calculated using Equation (3), roughly five shells of carbon atoms were obtained [61].

(3) $$n=\frac{\tau }{d}.$$

Using Equation (4), we previously calculated the total number of carbons per shell, for the oxidized carbon nano onion [61]:

(4) $$60\;\times \;n^{2},$$

#### 3.2.3. Nanocomposite Film Preparation

Nanocomposite films were prepared with a known method [56], with the components according to Table 2. PVA and CS 2% (wt %) in 2% (*v*/*v*) acetic acid were prepared. Dispersions of ox-CNO or CS-g-CNO (10 mg/mL) in 2% acetic acid were prepared using an ultrasonic bath (Branson, Madrid, Spain) for 2 h. All the components were mixed gently, according to Table 2.

The tensile test of the films was evaluated according to ASTM D6287, ASTM D618, and ASTM D882. Thicknesses were determined using a Mitutoyo digital micrometer No. 293-330 (Kawasaki, Japan), with three average values from each sample. The samples were placed in a desiccator at 10% relative humidity (RH) until the time of the test.

#### 3.2.4. Film Characterization

##### Fourier-Transform Infrared Spectroscopy (FTIR)

Chemical groups were identified by FTIR in the ATR mode (attenuated total reflectance) with a diamond tip (Shimadzu, Kyoto, Japan).

##### X-ray Diffraction (XRD)

The X-ray spectrum of the film structures was evaluated using the same equipment and the same parameters and equations described previously for CS-g-CNO.

##### Scanning Electron Microscope (SEM)

Surface morphology was studied by a scanning electron microscope (SEM; JEOL JSM-6490LA, Musashino, Tokyo, Japan). The voltage used was 20 kV with the mode of secondary backscattered electrons. For the proper conductivity of the samples, a coating of gold was prepared.

##### Tensile Strength of Films

Mechanical properties were tested using a universal SHIMADZU EZ-LZ test machine (Shimadzu, Kyoto, Japan), following the ASTM D882 standard. Six replicates per formulation were used. The gap between grips was 100 mm, the width of the film was 20 mm, and the test speed was 50 mm/min.

##### Thermal Studies

Thermal gravimetric analysis (TGA) was performed on a TA Instruments TGA Q50 V20.13 Build 39 (TA instrument, Delaware, New Castle, USA). Nanocomposite films were heated up to 1000 °C at a heating rate of 10 °C/min under air atmosphere (flow rate 80 mL/min). DSC cycle heating was performed between 25 and 350 °C and again cooling to 25 °C. The glass transition temperature (T_g_) was determined by differential scanning calorimetry using a DSC2A-00181 (TA instrument, Delaware, New Castle, USA) from the midpoint of the inflection tangent from the second heating at 10 °C/min. TGA and DSC data were analyzed using TA Instruments’ Universal Analysis software.

##### Degradation in Simulated Biological Fluid (SBF)

The hydrolytic degradation was carried out according to the previously reported procedure [56] based on the ASTM F1635-16 standard. Nanocomposite films were immersed in SBF (prepared as previously reported) at 37 °C for 25 days in a Memmert IN 110 incubator (Memmert, Schwabach, Germany) [56]. The weight loss (% W_l_) was calculated according to Equation (5).

(5) $$W_{l}\;\left(\%\right)=\frac{W_{0}-W_{d}}{W_{0}}\;\times 100.$$

The initial weight of the samples before immersion was W_0_, and the weight after drying for 48 h in the incubator at 37 °C was recorded as W_d_.

The water absorption was calculated by Equation (6). In all the experiments, a minimum of three samples was averaged:

(6) $$\%\;water\;absorption=\frac{W_{w}-W_{d}}{W_{d}}\;\times 100$$

The wet weight after immersion and surface drying with an absorbent paper was recorded as W_w,_ and the weight after drying for at least 72 h in the incubator at 37 °C registered as W_d._

The pH of the SBF was measured every day until the total test time was completed using an Accumet™ AB150 pH meter (Fisherbrand, Ottawa, Canada).

##### Biomodel In Vivo tests 

The biocompatibility in vivo of the nanocomposite films (F1–F5) was studied using Wistar rat subdermal tissue implantation [56,58,59]. Nanocomposite films of 5 mm of width and 10 mm of length were implanted in subdermal position according to ISO 10993-6. Commercial porcine collagen was used as a control. Wistar rats eight months old were supplied by the Bioterio of the Universidad del Valle. This research was reviewed, supported, and supervised by the Institutional Ethics Review Committee with experimental animals of the Universidad del Valle (CEAS 012-019).

After 30 days of implantation, the samples were recovered, fixed in buffered formalin, dehydrated in alcohol solutions of ascending concentration (70%, 80%, 95%, and 100%), diaphanized with xylol, and infiltrated with paraffin for later cutting to 4 µm. The samples were processed for histological analysis by hematoxylin and eosin (H&E) and Masson’s trichrome stain (MT) techniques.

##### Statistical Analysis

The analysis of variance (ANOVA) and the LSD method for mean separation, with a confidence level of 95% (α = 0.05), were used to evaluate the effect of the different formulations on the mechanical properties (Young’s modulus and Tensile strength). In vivo studies, mechanical properties and hydrolytic degradation are presented as mean values of at least three replicates ± SD. The Minitab 17 program was used for these statistical analyses.

## 4. Conclusions

The successful preparation of stable and compatible nanocomposite films based on CS/PVA/CS-g-CNO was confirmed from the mechanical, chemical, and thermal tests, as well as the subdermal implantation test. The thermal and mechanical stability was lower compared to composite films, including ox-CNO. Hydrolytic degradation in SBF of the films containing CS-g-CNO (F4 and F5) was higher, while water uptake and pH change demonstrating a stronger interaction with SBF compared to the composite films, including ox-CNO. The lower stability could be a result of higher CS content, which facilitates the hydrogen bonding with water, decreasing the water-resistant. Probably, a lower dispersion of CNO will occur due to the hydrogen bonding with CS/PVA chains, which was confirmed by SBF hydrolytic degradation and histological studies. For the hydrolytic degradation, samples with CS-g-CNO (F4 and F5) lost 14–16% of their initial weight after 25 days, confirming higher affinity with water. This result correlates well with the in vivo studies after 30 days of subdermal implantation in Wistar rats, where lower remaining material and higher biosorption for F4 and F5 were found but without any immune response. Carbon nano-onions grafting with chitosan improves the biocompatibility and reabsorption of the films, a fact that stimulates tissue regeneration. Upcoming studies will use in vitro and critical size defect analysis to determine the tissue regeneration capacity of CS/PVA/CS-g-CNO films.

## Figures and Tables

**Table 1:** Td_3%_, Young’s modulus, and glass transition temperature (T_g_) of the different film formulations (F1–F5).

Sample *	ox-CNO (wt %)	CS-g-CNO (wt %)	Td_3%_ (°C)	T_g_ (°C)	Young’s Modulus (GPa) **	Tensile Strength (MPa) **
F1	0	0	67.3	19.9	3.69 ± 0.45 ^a^	38.2 ± 2.88 ^a^
F2	0.50	0	50.8	80.2	3.60 ± 0.74 ^a^	38.3 ± 3.43 ^a^
F3	1.00	0	85.0	79.3	3.67 ± 0.77 ^a^	43.1 ± 3.23 ^a^
F4	0	0.50	78.3	51.6	1.36 ± 0.26 ^b^	23.7 ± 3.42 ^b^
F5	0	1.00	79.9	38.3	2.26 ± 0.15 ^c^	28.0 ± 2.27 ^b^

* Formulations: F1 (CS: PVA:ox-CNO 30.00:70.00:0); F2 (CS:PVA:ox-CNO 29.50:70.00:0.50); F3 (CS:PVA:ox-CNO 29.00:70.00:1.00); F4 (CS:PVA:CS-g-CNO 29.50:70.00:0.50); F5 (CS:PVA:CS-g-CNO 29.00:70.00:1.00). ** Values correspond to means ± standard deviation. Different superscript letters in the same column indicate significant differences (*p* < 0.05).

**Table 2:** Formulations used for the preparation of chitosan (CS)/poly (vinyl alcohol) (PVA)/CS-g-CNO or ox-CNO nanocomposite solutions.

Component	F1	F2	F3	F4	F5
CS (%)	30.00	29.50	29.00	29.50	29.00
PVA (%)	70.00	70.00	70.00	70.00	70.00
ox-CNO (%)	0	0.50	1.00	0	0
CS-g-CNO (%)	0	0	0	0.50	1.00

## Supplementary Materials

The following are available online, Figure S1. Mechanical properties of the films. (**A**) Young’s modulus and (**B**) Tensile strength of the different formulations: F1 (CS:PVA:ox-CNO 30.00:70.00:0); F2 (CS:PVA:ox-CNO 29.50:70.00:0.50); F3 (CS:PVA:OX-CNO 29.00:70.00:1.0); F4 (CS:PVA:CS-g-CNO 29.50:70.00:0.50); F5 (CS:PVA:CS-g-CNO 29.00:70.00:1.00). Figure S2. TGA curves of the formulations: F1 (CS:PVA:ox-CNO 30.00:70.00:0); F2 (CS:PVA:ox-CNO 29.50:70.00:0.50); F3 (CS:PVA:OX-CNO 29.00:70.00:1.0); F4 (CS:PVA:CS-g-CNO 29.50:70.00:0.50); F5 (CS:PVA:CS-g-CNO 29.00:70.00:1.00).

## References

[1] Hollister S.J. (2005). Porous scaffold design for tissue engineering. Nat. Mater..

[2] Chen G., Ushida T., Tateishi T. (2002). Scaffold design for tissue engineering. Macromol. Biosci..

[3] Eivazzadeh-Keihan R., Maleki A., de la Guardia M., Bani M.S., Chenab K.K., Pashazadeh-Panahi P., Baradaran B., Mokhtarzadeh A., Hamblin M.R. (2019). Carbon based nanomaterials for tissue engineering of bone: Building new bone on small black scaffolds: A review. J. Adv. Res..

[4] Harrison B.S., Atala A. (2007). Carbon nanotube applications for tissue engineering. Biomaterials.

[5] Gerasimenko A.Y., Ichkitidze L.P., Podgaetsky V.M., Selishchev S.V. (2015). Biomedical applications of promising nanomaterials with carbon nanotubes. Biomed. Eng..

[6] Shin S.R., Li Y.-C., Jang H.L., Khoshakhlagh P., Akbari M., Nasajpour A., Zhang Y.S., Tamayol A., Khademhosseini A. (2016). Graphene-based materials for tissue engineering. Adv. Drug Deliv. Rev..

[7] Yu X., Tang X., Gohil S.V., Laurencin C.T. (2015). Biomaterials for bone regenerative engineering. Adv. Healthc. Mater..

[8] Stratton S., Shelke N.B., Hoshino K., Rudraiah S., Kumbar S.G. (2016). Bioactive polymeric scaffolds for tissue engineering. Bioact. Mater..

[9] Dhandayuthapani B., Yoshida Y., Maekawa T., Kumar D.S. (2011). Polymeric scaffolds in tissue engineering application: A review. Int. J. Polym. Sci..

[10] Venkatesan J., Bhatnagar I., Manivasagan P., Kang K.-H., Kim S.-K. (2015). Alginate composites for bone tissue engineering: A review. Int. J. Biol. Macromol..

[11] Saravanan S., Leena R.S., Selvamurugan N. (2016). Chitosan based biocomposite scaffolds for bone tissue engineering. Int. J. Biol. Macromol..

[12] Niranjan R., Koushik C., Saravanan S., Moorthi A., Vairamani M., Selvamurugan N. (2013). A novel injectable temperature-sensitive zinc doped chitosan/β-glycerophosphate hydrogel for bone tissue engineering. Int. J. Biol. Macromol..

[13] Shui W., Zhang W., Yin L., Nan G., Liao Z., Zhang H., Wang N., Wu N., Chen X., Wen S. (2014). Characterization of scaffold carriers for BMP9-transduced osteoblastic progenitor cells in bone regeneration. J. Biomed. Mater. Res. Part. A.

[14] McFadden T.M., Duffy G.P., Allen A.B., Stevens H.Y., Schwarzmaier S.M., Plesnila N., Murphy J.M., Barry F.P., Guldberg R.E., O’brien F.J. (2013). The delayed addition of human mesenchymal stem cells to pre-formed endothelial cell networks results in functional vascularization of a collagen–glycosaminoglycan scaffold in vivo. Acta Biomater..

[15] Lin C.-Y., Chang Y.-H., Li K.-C., Lu C.-H., Sung L.-Y., Yeh C.-L., Lin K.-J., Huang S.-F., Yen T.-C., Hu Y.-C. (2013). The use of ASCs engineered to express BMP2 or TGF-β3 within scaffold constructs to promote calvarial bone repair. Biomaterials.

[16] Sun Y., Jiang Y., Liu Q., Gao T., Feng J.Q., Dechow P., D’Souza R.N., Qin C., Liu X. (2013). Biomimetic engineering of nanofibrous gelatin scaffolds with noncollagenous proteins for enhanced bone regeneration. Tissue Eng. Part. A..

[17] Saravanan S., Nethala S., Pattnaik S., Tripathi A., Moorthi A., Selvamurugan N. (2011). Preparation, characterization and antimicrobial activity of a bio-composite scaffold containing chitosan/nano-hydroxyapatite/nano-silver for bone tissue engineering. Int. J. Biol. Macromol..

[18] Khor E., Lim L.Y. (2003). Implantable applications of chitin and chitosan. Biomaterials.

[19] Soundarya S.P., Menon A.H., Chandran S.V., Selvamurugan N. (2018). Bone tissue engineering: Scaffold preparation using chitosan and other biomaterials with different design and fabrication techniques. Int. J. Biol. Macromol..

[20] Dhivya S., Keshav Narayan A., Logith Kumar R., Viji Chandran S., Vairamani M., Selvamurugan N. (2018). Proliferation and differentiation of mesenchymal stem cells on scaffolds containing chitosan, calcium polyphosphate and pigeonite for bone tissue engineering. Cell Prolif..

[21] Shamekhi M.A., Mirzadeh H., Mahdavi H., Rabiee A., Mohebbi-Kalhori D., Eslaminejad M.B. (2019). Graphene oxide containing chitosan scaffolds for cartilage tissue engineering. Int. J. Biol. Macromol..

[22] Kashi M., Baghbani F., Moztarzadeh F., Mobasheri H., Kowsari E. (2018). Green synthesis of degradable conductive thermosensitive oligopyrrole/chitosan hydrogel intended for cartilage tissue engineering. Int. J. Biol. Macromol..

[23] Ahmad M., Manzoor K., Ahmad S., Akram N., Ikram S. (2019). Chitosan-based nanocomposites for cardiac, liver, and wound healing applications. Applications of Nanocomposite Materials in Orthopedics.

[24] Wu G., Deng X., Song J., Chen F. (2018). Enhanced biological properties of biomimetic apatite fabricated polycaprolactone/chitosan nanofibrous bio-composite for tendon and ligament regeneration. J. Photochem. Photobiol. B Biol..

[25] Chen E., Yang L., Ye C., Zhang W., Ran J., Xue D., Wang Z., Pan Z., Hu Q. (2018). An asymmetric chitosan scaffold for tendon tissue engineering: In vitro and in vivo evaluation with rat tendon stem/progenitor cells. Acta Biomater..

[26] Qasim S., Zafar M., Najeeb S., Khurshid Z., Shah A., Husain S., Rehman I. (2018). Electrospinning of chitosan-based solutions for tissue engineering and regenerative medicine. Int. J. Mol. Sci..

[27] González-Quevedo D., Martínez-Medina I., Campos A., Campos F., Carriel V. (2018). Tissue engineering strategies for the treatment of tendon injuries: A systematic review and meta-analysis of animal models. Bone Jt. Res..

[28] Ueno H., Mori T., Fujinaga T. (2001). Topical formulations and wound healing applications of chitosan. Adv. Drug Deliv. Rev..

[29] Ratner B.D., Hoffman A.S., Schoen F.J., Lemons J.E. (2004). Biomaterials Science: An Introduction to Materials in Medicine.

[30] Thakur V.K., Voicu S.I. (2016). Recent advances in cellulose and chitosan based membranes for water purification: A concise review. Carbohydr. Polym..

[31] He Y., Miao J., Chen S., Zhang R., Zhang L., Tang H., Yang H. (2019). Preparation and characterization of a novel positively charged composite hollow fiber nanofiltration membrane based on chitosan lactate. Rsc Adv..

[32] Medina V.F., Griggs C.S., Mattei-Sosa J., Petery B., Gurtowski L. (2019). Advanced Filtration Membranes Using Chitosan and Graphene Oxide. U.S. Patent Application.

[33] Sun T., Guo X., Zhong R., Ma L., Li H., Gu Z., Guan J., Tan H., You C., Tian M. (2019). Interactions of oligochitosan with blood components. Int. J. Biol. Macromol..

[34] Heise K., Hobisch M., Sacarescu L., Maver U., Hobisch J., Reichelt T., Sega M., Fischer S., Spirk S. (2018). Low-molecular-weight sulfonated chitosan as template for anticoagulant nanoparticles. Int. J. Nanomed..

[35] Guo X., Sun T., Zhong R., Ma L., You C., Tian M., Li H., Wang C. (2018). Effects of chitosan oligosaccharides on human blood components. Front. Pharmacol..

[36] Dimassi S., Tabary N., Chai F., Blanchemain N., Martel B. (2018). Sulfonated and sulfated chitosan derivatives for biomedical applications: A review. Carbohydr. Polym..

[37] Mehta P., Al-Kinani A.A., Arshad M.S., Singh N., van der Merwe S.M., Chang M.-W., Alany R.G., Ahmad Z. (2019). Engineering and development of chitosan-based Nanocoatings for Ocular Contact Lenses. J. Pharm. Sci..

[38] Ali A., Ahmed S. (2018). A review on chitosan and its nanocomposites in drug delivery. Int. J. Biol. Macromol..

[39] Ahsan S.M., Thomas M., Reddy K.K., Sooraparaju S.G., Asthana A., Bhatnagar I. (2018). Chitosan as biomaterial in drug delivery and tissue engineering. Int. J. Biol. Macromol..

[40] Gomillion C.T. (2019). Assessing the potential of chitosan/polylactide nanoparticles for delivery of therapeutics for triple-negative breast cancer treatment. Regen. Eng. Transl. Med..

[41] Raval R., Rangnekar R.H., Raval K. Optimization of chitosan nanoparticles synthesis and its applications in fatty acid absorption. Materials, Energy and Environment Engineering.

[42] Berkland C., Qian J., Sullivan B.P. (2015). Micelle Sequestering Polymers. U.S. Patent Application.

[43] Hamedi H., Moradi S., Hudson S.M., Tonelli A.E. (2018). Chitosan based hydrogels and their applications for drug delivery in wound dressings: A review. Carbohydr. Polym..

[44] Mohebbi S., Nezhad M.N., Zarrintaj P., Jafari S.H., Gholizadeh S.S., Saeb M.R., Mozafari M. (2019). Chitosan in biomedical engineering: A critical review. Curr. Stem Cell Res. Ther..

[45] Cazón P., Vázquez M. (2019). Applications of Chitosan as Food Packaging Materials. Sustainable Agriculture Reviews 36.

[46] Wang H., Qian J., Ding F. (2018). Emerging chitosan-based films for food packaging applications. J. Agric. Food Chem..

[47] HPS A.K., Saurabh C.K., Adnan A.S., Fazita M.R.N., Syakir M.I., Davoudpour Y., Rafatullah M., Abdullah C.K., Haafiz M.K.M., Dungani R. (2016). A review on chitosan-cellulose blends and nanocellulose reinforced chitosan biocomposites: Properties and their applications. Carbohydr. Polym..

[48] Koosha M., Mirzadeh H., Shokrgozar M.A., Farokhi M. (2015). Nanoclay-reinforced electrospun chitosan/PVA nanocomposite nanofibers for biomedical applications. Rsc Adv..

[49] Umeyama T., Imahori H. (2012). Photofunctional hybrid nanocarbon materials. J. Phys. Chem. C.

[50] Rettenbacher A.S., Elliott B., Hudson J.S., Amirkhanian A., Echegoyen L. (2006). Preparation and functionalization of multilayer fullerenes (carbon nano-onions). Chem. Eur. J..

[51] Hirata A., Igarashi M., Kaito T. (2004). Study on solid lubricant properties of carbon onions produced by heat treatment of diamond clusters or particles. Tribol. Int..

[52] Ibáñez-Redín G., Furuta R.H.M., Wilson D., Shimizu F.M., Materon E.M., Arantes L.M.R.B., Melendez M.E., Carvalho A.L., Reis R.M., Chaur M.N. (2019). Screen-printed interdigitated electrodes modified with nanostructured carbon nano-onion films for detecting the cancer biomarker CA19-9. Mater. Sci. Eng. C.

[53] Ding L., Stilwell J., Zhang T., Elboudwarej O., Jiang H., Selegue J.P., Cooke P.A., Gray J.W., Chen F.F. (2005). Molecular characterization of the cytotoxic mechanism of multiwall carbon nanotubes and nano-onions on human skin fibroblast. Nano Lett..

[54] Fan J., Grande C.D., Rodrigues D.F. (2017). Biodegradation of graphene oxide-polymer nanocomposite films in wastewater. Environ. Sci. Nano.

[55] Grande C.D., Mangadlao J., Fan J., De Leon A., Delgado-Ospina J., Rojas J.G., Rodrigues D.F., Advincula R. (2017). Chitosan cross-linked graphene oxide nanocomposite films with antimicrobial activity for application in food industry. Macromol. Symp..

[56] Ruiz S., Tamayo A.J., Delgado Ospina J., Navia Porras P.D., Valencia Zapata E.M., Mina Hernandez H.J., Valencia H.C., Zuluaga F., Grande Tovar D.C. (2019). Antimicrobial films based on nanocomposites of chitosan/poly(vinyl alcohol)/graphene oxide for biomedical applications. Biomolecules.

[57] López Tenorio D., Valencia H.C., Valencia C., Zuluaga F., Valencia E.M., Mina H.J., Grande Tovar D.C. (2019). Evaluation of the biocompatibility of CS-Graphene oxide compounds in vivo. Int. J. Mol. Sci..

[58] Valencia C., Valencia C., Zuluaga F., Valencia M., Mina J., Grande-Tovar C. (2018). Synthesis and application of scaffolds of chitosan-graphene oxide by the freeze-drying method for tissue regeneration. Molecules.

[59] Tamayo Marín A.J., Londoño R.S., Delgado J., Navia Porras P.D., Valencia Zapata E.M., Mina Hernandez H.J., Valencia H.C., Grande Tovar D.C. (2019). Biocompatible and antimicrobial electrospun membranes based on nanocomposites of chitosan/poly (vinyl alcohol)/graphene oxide. Int. J. Mol. Sci..

[60] Valencia Zapata E.M., Mina Hernandez H.J., Grande Tovar D.C., Valencia Llano H.C., Diaz Escobar A.J., Vázquez-Lasa B., San Román J., Rojo L. (2019). Novel bioactive and antibacterial acrylic bone cement nanocomposites modified with graphene oxide and chitosan. Int. J. Mol. Sci..

[61] Grande Tovar C.D., Castro J.I., Valencia C.H., Navia Porras D.P., Hernandez M., Herminsul J., Valencia M.E., Velásquez J.D., Chaur M.N. (2019). Preparation of chitosan/poly (vinyl alcohol) nanocomposite films incorporated with oxidized carbon nano-onions (multi-layer fullerenes) for tissue-engineering applications. Biomolecules.

[62] Wu Z., Feng W., Feng Y., Liu Q., Xu X., Sekino T., Fujii A., Ozaki M. (2007). Preparation and characterization of chitosan-grafted multiwalled carbon nanotubes and their electrochemical properties. Carbon.

[63] Osswald S., Havel M., Gogotsi Y. (2007). Monitoring oxidation of multiwalled carbon nanotubes by Raman spectroscopy. J. Raman Spectrosc..

[64] Shriner R.L., Fuson R.C., Curtin D.Y. (1948). The systematic identification of organic compounds.

[65] Ke G., Guan W., Tang C., Guan W., Zeng D., Deng F. (2007). Covalent functionalization of multiwalled carbon nanotubes with a low molecular weight chitosan. Biomacromolecules.

[66] Mallakpour S., Zadehnazari A. (2014). A facile, efficient, and rapid covalent functionalization of multi-walled carbon nanotubes with natural amino acids under microwave irradiation. Prog. Org. Coat..

[67] Chattopadhyay J., Mukherjee A., Chakraborty S., Kang J., Loos P.J., Kelly K.F., Schmidt H.K., Billups W.E. (2009). Exfoliated soluble graphite. Carbon.

[68] Bustos-Ramírez K., Martínez-Hernández A.L., Martínez-Barrera G., Icaza M.D., Castaño V.M., Velasco-Santos C. (2013). Covalently bonded chitosan on graphene oxide via redox reaction. Materials.

[69] Cioffi C.T., Palkar A., Melin F., Kumbhar A., Echegoyen L., Melle-Franco M., Zerbetto F., Rahman G.M.A., Ehli C., Sgobba V. (2009). A carbon nano-onion–ferrocene donor–acceptor system: Synthesis, characterization and properties. Chem. Eur. J..

[70] Carson L., Kelly-Brown C., Stewart M., Oki A., Regisford G., Luo Z., Bakhmutov V.I. (2009). Synthesis and characterization of chitosan–carbon nanotube composites. Mater. Lett..

[71] Srinivasa P.C., Ramesh M.N., Kumar K.R., Tharanathan R.N. (2003). Properties and sorption studies of chitosan–polyvinyl alcohol blend films. Carbohydr. Polym..

[72] Pandele A.M., Ionita M., Crica L., Dinescu S., Costache M., Iovu H. (2014). Synthesis, characterization, and in vitro studies of graphene oxide/chitosan-polyvinyl alcohol films. Carbohydr. Polym..

[73] Jia Y.-T., Gong J., Gu X.-H., Kim H.-Y., Dong J., Shen X.-Y. (2007). Fabrication and characterization of poly (vinyl alcohol)/chitosan blend nanofibers produced by electrospinning method. Carbohydr. Polym..

[74] Lu L., Peng F., Jiang Z., Wang J. (2006). Poly(vinyl alcohol)/chitosan blend membranes for pervaporation of benzene/cyclohexane mixtures. J. Appl. Polym. Sci..

[75] Yadav I., Nayak S.K., Rathnam V.S.S., Banerjee I., Ray S.S., Anis A., Pal K. (2018). Reinforcing effect of graphene oxide reinforcement on the properties of poly (vinyl alcohol) and carboxymethyl tamarind gum based phase-separated film. J. Mech. Behav. Biomed. Mater..

[76] Yang X., Tu Y., Li L., Shang S., Tao X. (2010). Well-dispersed chitosan/graphene oxide nanocomposites. Acs Appl. Mater. Interfaces.

[77] Espigares I., Elvira C., Mano J.F., Vázquez B., San Román J., Reis R.L. (2002). New partially degradable and bioactive acrylic bone cements based on starch blends and ceramic fillers. Biomaterials.

[78] Herath H.M.T.U., Di Silvio L., Evans J.R.G. (2010). Biological evaluation of solid freeformed, hard tissue scaffolds for orthopedic applications. J. Appl. Biomater. Biomech..

[79] Figueira Maldonado E. (2008). Degradación hidrolítica a diferentes pH de un material compuesto Poli(ácido láctico)/Quitosano, Proyecto de grado.

[80] Depan D., Shah J.S., Misra R.D.K. (2013). Degradation mechanism and increased stability of chitosan-based hybrid scaffolds cross-linked with nanostructured carbon: Process-structure-functional property relationship. Polym. Degrad. Stab..

[81] Maruyama M., Ito M. (1996). In vitro properties of a chitosan-bonded self-hardening paste with hydroxyapatite granules. J. Biomed. Mater. Res..

[82] Tomihata K., Ikada Y. (1997). In vitro and in vivo degradation of films of chitin and its deacetylated derivatives. Biomaterials.

[83] Pella M.C.G., Lima-Tenório M.K., Tenorio-Neto E.T., Guilherme M.R., Muniz E.C., Rubira A.F. (2018). Chitosan-based hydrogels: From preparation to biomedical applications. Carbohydr. Polym..

[84] Fujita M., Ishihara M., Simizu M., Obara K., Ishizuka T., Saito Y., Yura H., Morimoto Y., Takase B., Matsui T. (2004). Vascularization in vivo caused by the controlled release of fibroblast growth factor-2 from an injectable chitosan/non-anticoagulant heparin hydrogel. Biomaterials.

[85] Sok V., Fragoso A. (2018). Preparation and characterization of alkaline phosphatase, horseradish peroxidase, and glucose oxidase conjugates with carboxylated carbon nano-onions. Prep. Biochem. Biotechnol..

[86] Vatanpour V., Safarpour M., Khataee A., Zarrabi H., Yekavalangi M.E., Kavian M. (2017). A thin film nanocomposite reverse osmosis membrane containing amine-functionalized carbon nanotubes. Sep. Purif. Technol..

