# Supplementary material for: Nanocomposite Films of Chitosan-Grafted Carbon Nano-Onions for Biomedical Applications
Source: Molecules. 2020 Mar 7;25(5):1203. doi: 10.3390/molecules25051203 (PMC7179466; doi:10.3390/molecules25051203)
Supplement: Supplementary file 1 [file molecules-25-01203-s001.pdf]

Supporting file

Nanocomposite Films of Chitosan-Grafted Carbon Nano-Onions for Biomedical Applications

Carlos David Grande Tovar [1], Jorge Iván Castro [2], Carlos Humberto Valencia [3], Diana Paola Navia Porras [4], José Herminsul Mina Hernandez [5,*], Mayra Eliana Valencia [5] and Manuel N. Chaur [2,6,*]

1. Grupo de investigación de fotoquímica y fotobiología, Universidad del Atlántico, Carrera 30 Número 8-49, Puerto Colombia 081008, Colombia; carlosgrande@mail.uniatlantico.edu.co
2. Grupo de Investigación SIMERQO, Departamento de Química, Universidad del Valle, Calle 13 No. 100-00, Cali 76001, Colombia; jorge.castro@correounivalle.edu.co (J.I.C.);
3. Escuela de Odontología, Grupo biomateriales dentales, Universidad del Valle, Calle 13 No. 100-00, Cali 76001, Colombia; carlos.humberto.valencia@correounivalle.edu.co
4. Grupo de Investigación Biotecnología, Facultad de Ingeniería, Universidad de San Buenaventura Cali, Carrera 122 # 6-65, Cali 76001, Colombia; dpnavia@usbcali.edu.co
5. Escuela de Ingeniería de Materiales, Facultad de Ingeniería, Universidad del Valle, Calle 13 No. 100-00, Santiago de Cali 760032, Colombia; valencia.mayra@correounivalle.edu.co
6. Centro de Excelencia en Nuevos Materiales (CENM), Universidad del Valle, Calle 13 No. 100-00, Santiago de Cali 760032, Colombia

* Correspondence: jose.mina@correounivalle.edu.co (J.H.M.H.); manuel.chaur@correounivalle.edu.co (M.N.C.)

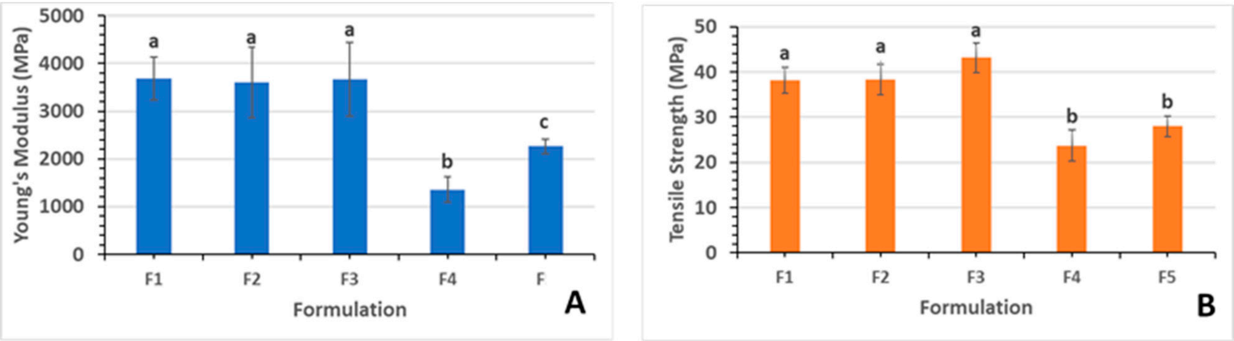

Figure S1. Mechanical properties of the films. A) Young's modulus and B) Tensile strength of the different formulations: F1 (CS:PVA:ox-CNO 30.00:70.00:0); F2 (CS:PVA:ox-CNO 29.50:70.00:0.50); F3 (CS:PVA:OX-CNO 29.00:70.00:1.0); F4 (CS:PVA:CS-g-CNO 29.50:70.00:0.50); F5 (CS:PVA:CS-g-CNO 29.00:70.00:1.00).

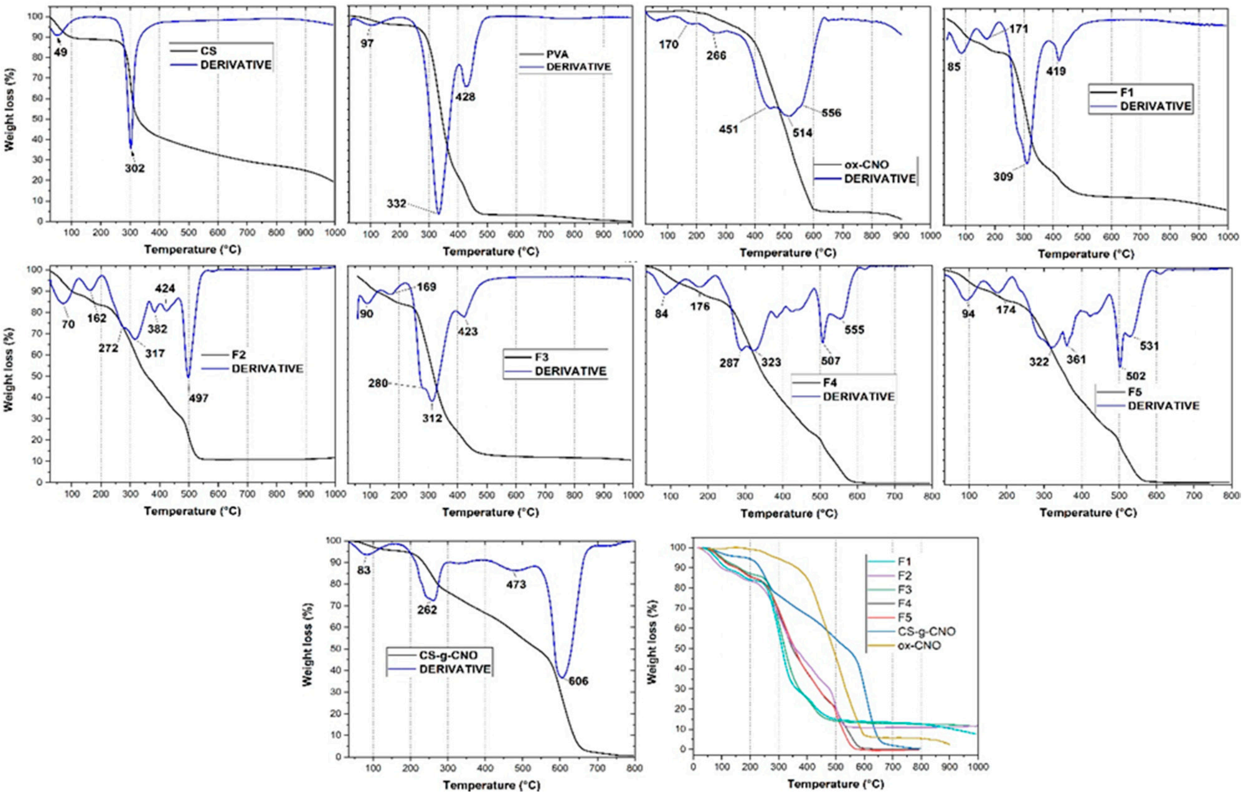

Figure S2. TGA curves of the formulations: F1 (CS:PVA:ox-CNO 30.00:70.00:0); F2 (CS:PVA:ox-CNO 29.50:70.00:0.50); F3 (CS:PVA:OX-CNO 29.00:70.00:1.0); F4 (CS:PVA:CS-g-CNO 29.50:70.00:0.50); F5 (CS:PVA:CS-g-CNO 29.00:70.00:1.00).
